# Women’s perspectives on disrespect and abuse during facility-based childbirth in Ethiopia: a qualitative study

**DOI:** 10.1186/s12884-023-05762-8

**Published:** 2023-06-14

**Authors:** Yohannes Mehretie Adinew, Janet Kelly, Morgan Smith, Amy Marshall

**Affiliations:** 1 Adelaide Nursing School, The University of Adelaide, Adelaide, Australia; 2 College of Health sciences and Medicine, Wolaita Sodo University, Sodo, Ethiopia; 3 College of Nursing and Health Sciences, Flinders University, Adelaide, Australia

**Keywords:** Women, Human rights abuses, Respect, Birthing centers, Ethiopia

## Abstract

**Background:**

Disrespect and abuse violates women’s basic human rights and autonomy and can traumatize women who are already in a vulnerable position during childbirth and deter them from utilizing skilled care for future childbirth. This study explored women’s perspectives on the acceptability of disrespect and abuse during facility-based childbirth in Ethiopia.

**Methods:**

A qualitative descriptive design using five focus group discussions and fifteen in-depth, semi-structured, interviews was conducted with women between October 2019 to January 2020 in north Showa zone of Oromia region, central Ethiopia. Using purposive sampling, women who had given birth at public health facilities of North Showa zone during the twelve months preceding data collection were recruited, regardless of birth outcome. Inductive thematic analysis using Open Code software was used to explore the perspectives of participants.

**Results:**

While women reject disrespectful and abusive acts during childbirth generally, they may consider some disrespectful acts as acceptable and or necessary under certain circumstances. Four emerging themes were identified. (1) Disrespect and abuse is not acceptable, (2) Disrespectful and abusive actions are acceptable only if intended to save lives, (3) Disrespectful and abusive actions are an accepted part of everyday practice to prevent complications and adverse outcomes, (4) Disrespectful and abusive actions are necessary to discipline disobedient women.

**Conclusion:**

Women’s perceptions of disrespectful and abusive acts of care providers is deeply rooted within the context of violence in Ethiopia and the societal hierarchies that have systematically disempowered women. Given the pervasiveness of disrespect and abusive actions during childbirth, policymakers, clinical managers and care providers must take these essential contextual and societal norms into account and devise comprehensive clinical interventions that addresses the root causes.

## Background

Childbirth is one of the most important and memorable events in the lives of women [1]. For women in developing countries however, pregnancy and childbirth places women at a substantial risk of suffering, ill-health and even death [2]. Complications from pregnancy and childbirth are the leading causes of mortality and morbidity for women of childbearing age in developing nations [3]. To reduce maternal and neonatal mortality, low resource countries are encouraging women to give birth in a health facility with the assistance of a skilled birth attendant. This is considered the most effective and cost-efficient strategy [4].

Globally, there is recognition that every woman has the right to the highest attainable standard of health, which includes the right to quality and respectful maternity care during childbirth [5, 6]. Respectful maternity care (RMC) has been defined by World Health Organization (WHO) as “care organized for and provided to all women in a manner that maintains their dignity, privacy, and confidentiality, ensures freedom from harm and abuse, and enables informed choice, and continuous support during labor and childbirth” [7]. Violations of these principles has been recognized as a priority issue by the WHO in improving maternity care quality and utilization [5].

Previous studies identified that women’s experiences of disrespectful and abusive care while in childbirth facilities has significantly impacted their utilization of these services [8–12]. Some women opt to travel to distant health facilities in search of quality service [13, 14], indicating that disrespectful care is a powerful deterrent to seeking skilled birth care together with geographic inaccessibility and financial constraints [15].

Evidence suggests that many women in Ethiopia may experience disrespect and abuse during childbirth [16, 17]. Women themselves report that poor provider attitudes influences their use of maternal health services and that fear of disrespect and abuse is discouraging them from seeking lifesaving maternity care at health facilities [18]. Reducing maternal morbidity and mortality remains a key health challenge for Ethiopia where 401 women died per 100,000 livebirths, in 2017 [19]. As a result of the increasing need of client-centered care, Ethiopia has incorporated compassionate and respectful maternity care in its latest health sector strategic plan [20].

There has been a growing consensus over time that client’s perspectives of the maternity care they receive in facilities are critical for maintaining and monitoring the quality of health care [21–23] Exploring women’s perspectives enables the strengths and gaps in care to be identified, inform more responsive and culturally acceptable care, leading to an increase in service utilization and better health care outcomes [24]. Studies have identified that satisfied clients are more likely to return in the future [24], adhere to care provider’s recommendations [25], and recommend the institution to their friends and relatives, effecting an increased demand for the service [26].

Community-based studies that report on women’s perspectives on disrespectful and abusive care are still lacking in Ethiopia. Most previous studies were conducted in health facilities, where social desirability bias and fear of retaliatory action could potentially influence women’s response. Therefore, this community-based study aimed to explore how women perceive health care professionals’ conduct that could be classified as disrespect and abuse by an independent observer, researcher, or advocate, in order to bring women’s voices into the maternal health quality improvement agenda.

## Methods

### Study design

Interviews and focus group discussions were conducted with women as part of a larger mixed methods study that examined disrespect and abuse of women during facility-based childbirth in Ethiopia from the perspectives of women and health care providers. A qualitative description design [27, 28] was used to investigate women’s perspectives of disrespect and abuse in public facility based birthing services. The perspectives of care providers are reported in a separate paper [29].

### Study setting

This study was conducted in north Showa zone of Oromia region, central Ethiopia, located 110 km north of the capital Addis Ababa. The total population is approximately 1.5 million and 48% are women [30]. Afan Oromo and Amharic are the main languages spoken. There are three hospitals, 62 health centers and 268 health posts in this zone.

### Recruitment of study participants

Participants were women who had given birth at public health facilities of North Showa zone during the twelve months preceding the study, regardless of the birth outcome. Women who gave birth at home, those who were acutely physically or mentally unwell and those with a disability that would prevent them talking to a researcher were excluded. A list, including contact details, of women who gave birth at public health facility is maintained by health extension workers. Initial recruitment and information about the study was provided by health extension workers, when visiting women in their homes. Following informed consent interested women then choose whether to be involved in a semi structured, in depth, interview (IDI) or a focus group discussion (FGD), at a time and place of their choice, but not both. Health extension workers work at health posts and do not have any involvement in facility-based birthing care. Purposive sampling was used to include adult participants across a range of ages, parity, and economic circumstances. The FGDs were grouped by urbanicity to maintain homogeneity, and to reflect perceptions of participants from urban and rural settings. Information saturation (when ideas started to be repeated and no more new ideas emerged) was used to determine the final number of IDIs and FGDs. Six to eight women participated in each FGD making the total FGD participants 39. The 15 in-depth interview participants raised the total number of study participants to 54.

### Patient and public involvement

No patient involved.

### Data collection tool and procedures

The research team consisting of a PhD student and three supervisors utilized the findings of a systematic review [31] to develop scenarios of different types of disrespect and abuse women may experience during childbirth in Ethiopia [32]. A prioritization exercise was conducted in Ethiopia among women in an adjacent district not included in the study to ensure these scenarios were understandable, clear and concise, and accessible and understandable for potential participants from a variety of different cultural and geographical backgrounds. Nine scenarios were developed **(see** Table 1**)**. The interviews were conducted by the primary author. The FGDs were facilitated by two people – the primary author moderated the discussion by asking participants to respond to open-ended questions and a second person (the research assistant note-taker) took detailed notes on the discussion.

**Table 1:** Sample Disrespect and Abuse questions

No.	Questions
1.	If a woman was pinched or slapped by a health worker during her childbirth, would this be acceptable? When would it be acceptable?
2.	If a woman was yelled or shouted at by a health worker during her childbirth, would this be acceptable? When would it be acceptable?
3.	If a health worker was mean and refused to help a woman during her delivery, would this be acceptable? When would it be acceptable?
4.	If a health worker physically held a woman down during her childbirth, would this be acceptable? When would it be acceptable?
5.	If a health worker threatens a woman by unfavorable procedure like CS or referral or bad outcome for her or her baby during her childbirth, would this be acceptable? When would it be acceptable?
6.	If a health worker disallowed a woman to deliver in a position of her choice during her childbirth, would this be acceptable? When would it be acceptable?
7.	If a health worker performs a procedure without getting consent during her childbirth, would this be acceptable? When would it be acceptable?
8.	If a health worker forcefully opens a woman’s leg during her childbirth, would this be acceptable? When would it be acceptable?
9.	If a health worker disallowed a woman birth companion during her childbirth, would this be acceptable? When would it be acceptable?

During interviews and focus groups, participants were presented with each scenario and asked when, if ever, this scenario would be acceptable. Women were invited to reflect on the ‘acceptability’ of each hypothetical disrespect and abuse during childbirth generally, rather than being asked to disclose personal instances of disrespectful and abusive treatment. This approach aimed to prevent re-traumatising women or expecting them to share highly personal information in a group setting.

Fifteen in depth interviews (IDIs) and five focus group discussions (FGDs) were concurrently conducted from 5 to 2019 to 25 January 2020. Individual participants were interviewed in their homes or another preferred location and were accompanied by a person of their choice. Interviews lasted approximately 50 min. Focus groups took place in community settings and lasted about 180 min. Care was taken to maintain confidentiality of study participants by allocating interview and focus group codes and maintaining privacy during interviews. The interviews and focus groups were audio (voice) recorded with permission, with field memos also taken.

### Data analysis

IDIs and FGDs were conducted in a local language (Afan Oromo or Amharic) by the primary author, translated and transcribed simultaneously by the primary author and reviewed by another Ethiopian based bilingual researcher. De-identified transcripts were stored on a password protected computer. An inductive thematic analysis approach as described by Braun and Clarke (2006) was used to identify key themes, richly describe large bodies of qualitative data and highlight similarities and differences in experiences [33]. Open coding of transcripts was performed line-by-line. Codes were then analysed alongside field memos. Themes were identified and representative quotes were selected to illustrate the range of voices in each theme.

Reliability testing was conducted in two stages: (1) two Ethiopian researchers jointly coded five transcripts and (2) then independently coded four transcripts and discussed coding decisions until consensus. A subset of the coded transcripts was reviewed by another independent Ethiopian researcher to check reliability of the coding. Open Code software version 4.0.2.3 was used for the data analysis. This paper is reported according to the consolidated criteria for reporting qualitative research (COREQ) [34].

## Results

### Views regarding acceptability of disrespect and abuse during facility-based childbirth

While women generally considered disrespectful and abusive acts during childbirth as unacceptable, some disrespectful acts in the scenarios presented were considered acceptable or necessary by the women, under certain circumstances. Women did not consistently hold the same viewpoint across all scenarios, rather they offered different perspectives depending on each scenario. Women’s responses and perspectives are presented under four main themes.

**Fig. 1:**
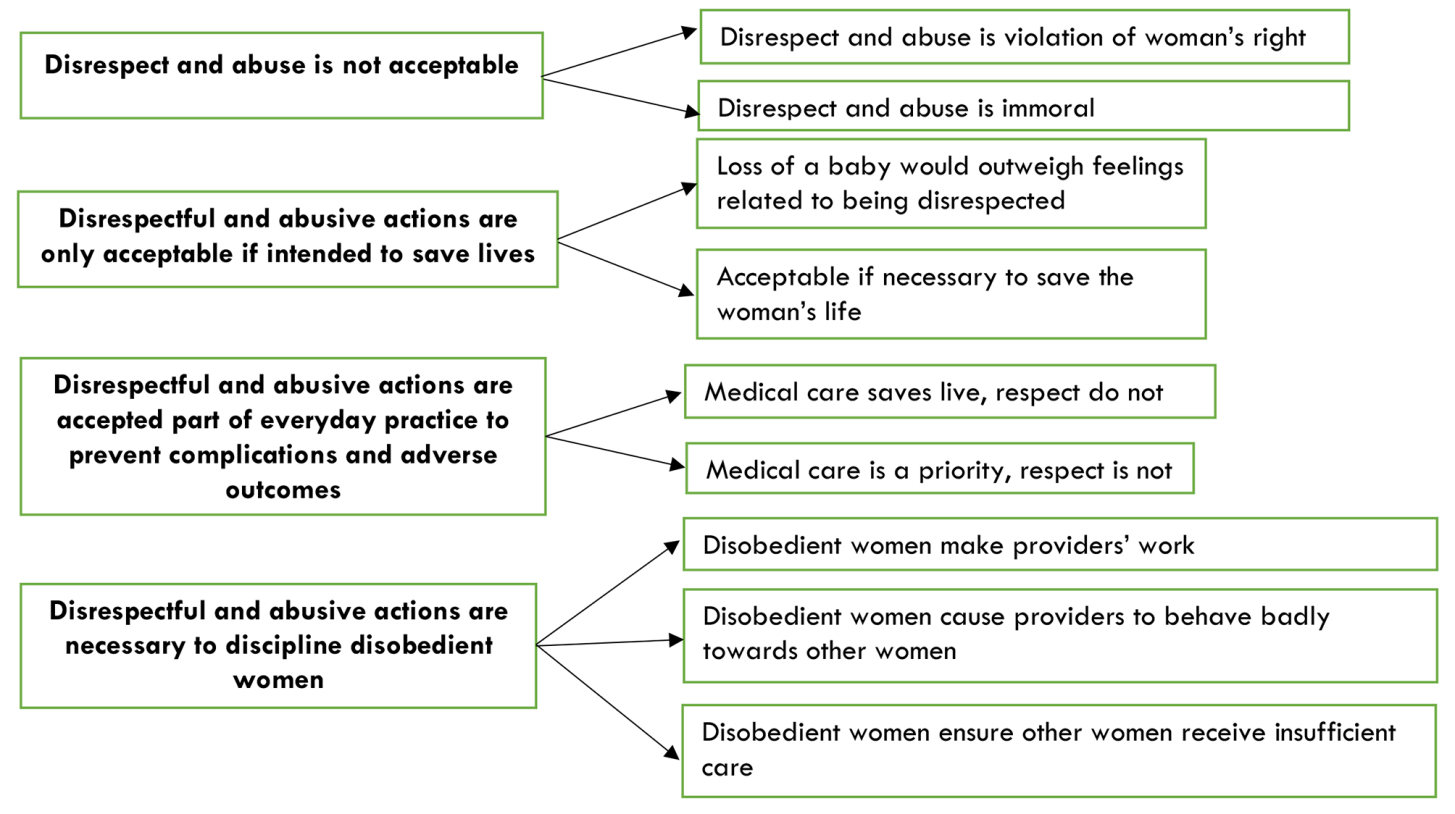
Schematic presentation of the identified themes and subthemes

The four emerging themes are: (1) Disrespect and abuse is not acceptable, (2) Disrespectful and abusive actions are sometimes acceptable if intended to save lives, (3) Disrespectful and abusive actions are an accepted part of everyday practice to prevent complications and adverse outcomes, (4) Disrespectful and abusive actions are necessary to discipline disobedient women. Figure 1 shows the identified themes.

### Disrespect and abuse is not acceptable

For some of the participants physical and verbal abuse, failure to seek consent and denial of care were considered breeches of a woman’s rights. In some instances, such actions were considered immoral. These disrespectful and abusive actions were never considered acceptable.

### Disrespect and abuse is violation of woman’s right

#### Physical abuse

Participants were invited to reflect on acceptability of women being slapped or pinched by providers during childbirth. Most participants agreed that pinching or slapping laboring women could never be justified. Regardless of how a woman behaves or how busy the provider is, pinching or slapping a laboring woman was considered to be unacceptable and a violation of her rights.

*“No reason can justify slapping of a laboring woman. It is a clear violation of woman’s right and have to be actively discouraged.” (Urban FGD)*

Forcefully holding down women onto the delivery bed was another scenario presented to the women and some women considered this an abuse of women’s freedom of movement. These participants believed that a woman should be free to leave the delivery couch if she wanted to do so.

*“Women should be allowed to move around. Holding them down against their will violates their right.”* (Rural IDI)

When asked if it is acceptable for providers to forcefully open women’s legs in order to assist them, some participants identified that women may refuse to open their legs due to fear of disclosing their most private body parts to providers, or they may spontaneously close their legs during labor due to pain. Most participants considered forcefully opening women’s legs unacceptable for any reason, even to save life. One woman in a rural IDI explained,

*“It is never acceptable to forcefully open a woman’s legs even if it is to save her baby. The only thing the provider can do is to tell her the importance of opening her leg. If she does not comply, it is better to involve her family to convince her than forcefully opening her leg. It is against her rights.”* (Rural IDI)

### Verbal abuse

Some participants considered providers’ yelling or shouting at a laboring woman as unacceptable, irrespective of the intention behind the act. They believed that care providers must not raise their voice when they communicate with a laboring woman, and providers who yell or shout at women lack self-control and should not be involved in birthing care. One participant from an urban area said;

*“Raising a voice to someone is a sign of disrespect and a laboring mother does not deserve it. It is never acceptable for a provider to yell or shout at a laboring woman. A provider who has no respect for a woman should not be allowed in the birthing care.”* (Urban IDI)

Another example presented to the women participants was the acceptability or non-acceptability of threatening women with an unfavorable procedure, or referral or predicting a bad outcome for her or her baby. Some participants considered it unacceptable for a health care provider to threaten women under any circumstance. These participants believed that threatening a woman is not part of the care the women came for and therefore should not be practiced.

*“It is totally unacceptable to threaten a laboring woman for whatever reason. Women visit health facility to receive care not a threat. I believe it is counterproductive and violates her rights.”* (Urban IDI)

Performing a procedure without obtaining consent was another example of disrespect and abuse presented to participants to discuss. The majority of participants considered this unacceptable. They believed that consent must be sought prior to any procedure despite its medical indication and emergency nature of the situation.

*“No women must undergo any procedure without her informed consent. Women have the right to know and willingly consent to or refuse a procedure. The procedure might be important, but her consent is necessary.”* (Urban IDI)

Participants were invited to reflect on whether disallowing of a birthing companion is acceptable, and the majority considered it unacceptable. They stated that having a birthing companion is a right of every childbearing woman and disallowing is a violation of her rights.

*“Women have the right to be accompanied by person of their choice during childbirth and denying them a birth companion is unacceptable and violation of their basic rights.”* (Urban FGD)

Participants were asked if it is acceptable for a health worker to refuse to help a woman during childbirth. No participant considered refusal of care acceptable for any reason and under any circumstance. The majority of participants not only rejected withholding or refusal of care but also condemned it.

*“Withholding care is a murder, I do not see any difference in between. It is an act of evil, no decent human can do so. It is a gross crime to refuse assisting a laboring woman in a facility.”* (Urban IDI)

Participants reflected the same view on disallowance of preferred birthing position, no participant considered it acceptable. Even if most of the participants prefer the traditional lithotomy position for childbirth, they believed that women who prefer other positions must be supported.

*“It is women’s right to choose a birthing position suitable to them. The role of the provider is to assist accordingly, not disallowing them their rights.”* (Urban IDI)

### Disrespect and abuse is immoral

For some participants the presented scenarios were not only unacceptable, but they were also viewed as immoral. In relation to whether it is acceptable for a care provider to slap or pinch a woman during childbirth, participants explained that it is immoral for a care provider to raise hand on a laboring woman. According to participants, care providers are supposed to behave and do better than abusing a birthing woman. Similarly, there were participants who expressed a viewpoint that shouting and yelling are morally unacceptable. They questioned the moral value of providers who do so. According to these participants, such behavior does not go with moral values of professionalism. Threatening women with an unfavorable procedure, or referral or predicting a bad outcome for her or her baby was also considered morally unacceptable behavior among some participants. Similarly, participants were invited to reflect their views on acceptability of forcefully holding women to the delivery bed and withholding care. Some participants were disgusted with the idea that care providers can possibly deny women the care they came for. For these participants, denial of care is not acceptable under any circumstance and immoral above anything else. FGD participants from setting explained that:

*“It is unfair and immoral for a provider to get in to conflict with a laboring woman and deny her care.”* (Rural FGD)

### Disrespectful and abusive actions are only acceptable if intended to save lives

Some participants considered that some of the care provider’s behaviors presented in the scenarios may be acceptable if the intention were to save the life of the woman and or her baby.

For these participants, the intention of the provider and the level of threat to the lives of the mother and baby determines the acceptability or unacceptability of the action undertaken by health care providers. The action has to contribute to safe delivery to be considered acceptable.

### Loss of a baby would outweigh feelings related to being disrespected

Some participants considered the presented scenario may be acceptable if the intention were to save life. These participants focused on the reason or intention behind the behaviour and perceived those complications in labour and potential loss of a baby would outweigh feelings related to disrespect or pain a woman may feel by being slapped or pinched.

*“It is ok if a provider pinches or slaps a woman on her thigh and encourage her to push. Developing complications or loss of a baby is more painful than being slapped or pinched. What is not acceptable is slapping a woman as a punishment.” (*Rural IDI*)*

Similarly, some participants considered that forcefully opening women’s legs could be acceptable if it was necessary to save the baby’s life. They were concerned that the baby may be suffocated if the mother closes her legs while the baby’s head is engaged.

*“I consider forcefully opening woman’s legs acceptable only if it is an act to save her child. It is nothing compared to lose of a baby. If they [providers] use force, it must be for the good of the mother.”* (Urban FGD)

### Acceptable if necessary to save the woman’s life

Likewise, participants expressed a viewpoint that the only time verbal threatening may be considered acceptable was if it is done to save life of the mother as well as her baby.

*“Threatening of a woman is acceptable only if it is aimed to save the life of the mother or her baby. Otherwise, it is unacceptable to threaten women.”* (Rural FGD)

Some women also considered performing a procedure without first getting consent was acceptable if it were to save lives. These participants considered that emergency situations may not give providers sufficient time to explain procedures and obtain consent, rather the woman’s circumstance may compel them to rush to life saving procedure.

*“Performing a procedure without getting consent is acceptable in case of emergency. Provider’s primary aim is to save the woman and her baby.”* (Rural IDI)

### Disrespectful and abusive actions are an accepted part of everyday practice to prevent potential complications and adverse outcomes

A large number of participants from both rural and urban areas considered that the presented examples may be a necessary everyday part of providing care, and that health professionals employ these strategies to prevent potential adverse outcomes.

#### Medical care saves live, respect does not

For some women, forcefully holding a woman to a delivery bed is considered an acceptable and potentially a lifesaving act. This was based on a belief that if a woman leaves the bed, she may suffer awful consequences.

*“Why would a woman leave the delivery bed once she started pushing? She may lose her baby to the ground and experience genital tear. The provider in charge has to forcefully hold her down to the delivery bed.”* (Rural IDI*)*

A few participants proposed that forcefully opening a women’s legs was not only necessary to save a baby’s life, but also an acceptable behavior due to the authority and knowledge of the care provider. These participants discussed that women must comply with providers orders once they are on the delivery couch because the providers know what works for the women. Thus, use of force to open women’s legs is considered necessary to save the baby.

*“I consider forcefully opening women’s legs* necessary. Because c*losing legs during labor could kill the baby. Thus, providers have to forcefully open women’s legs and takeout the baby”* (Rural FGD)

The majority of the participants normalized providers’ yelling or shouting at a woman as an act to save life. They perceived that providers raise their voice to warn the woman so that she can concentrate and comply with their orders. Some expressed a viewpoint that if the provider does not raise their voice, then the woman may not understand the seriousness of the situation.

*“Providers raise voice to make woman actively comply with orders and avoid complications. It is for the good of the mother, it saves life.”* (Rural FGD)

Similarly, some participants considered threatening women was acceptable and that women would not suffer negatively from these threats, but rather may benefit when taking the threats seriously. They considered threatening is like an alarm that alerts women to danger.

*“Threatening has no harm, rather it makes woman more focused and escape possible complications. Providers threaten women to avoid unforeseen problems and I consider it saves life.”* (Rural IDI)

#### Medical care is a priority, respect is not

Some participants considered that a provider can perform a procedure if it is medically indicated, without the woman’s consent. These participants perceived that medical indication for a procedure could override the need for woman’s consent, based on a belief that providers know best what is needed for the woman, and that her consent is a lower priority.

*“The provider is a professional who has the knowledge to do the right thing and can decide what to do and when. The woman [does not have this knowledge], and her opinion and consent are not a priority.”* (Rural FGD)

### Disrespectful and abusive actions are necessary to discipline disobedient women

Some participants discussed the scenarios and what they considered to be acceptable or expected behaviors in relation to the workload of care providers, and the implications for other birthing women if one woman was [perceived to be] disruptive or disobedient.

#### Disobedient women make providers’ work difficult

Some participants discussed that some women may ask too many questions, argue with providers or refuse a procedure. They perceived these women’s behaviors could be viewed as difficult, uncooperative, and/or disobedient and that slapping or pinching them in these circumstances may be necessary. Upon further enquiry, participants offered an explanation that “disobedient” women irresponsibly put their and the baby’s lives at risk. They felt that health care providers’ primary responsibility is to deliver a live baby to a healthy mother and if women are uncooperative, the health care provider may be justified to slap or pinch the woman.

*“Some women are disobedient and do not listen to the providers. They unnecessarily put their and the baby’s life at risk. So, it is acceptable to slap, or pinch disobedient women to discipline them.”* (Urban IDI)

Some women empathized with care providers’ heavy workload and that some women’s behavior makes their work more difficult.

*“Providers are human beings and deserve respect and it irritates me when some women look down on them or make their job more difficult. Such unsympathetic and inconsiderate women deserve a slap. I wish I could slap them myself.”* (Rural IDI)

#### Disobedient women cause providers to behave badly towards other women

Some participants were also concerned that uncooperative women could test providers’ patience, and annoy them by asking too many questions, and this could then negatively impact health care providers approach towards other women.

*“Sometimes providers could ignore other innocent women in retaliation, only because one rude woman has annoyed them by asking question. I understand providers have feelings too, but they better slap that specific woman [rather] than neglecting others.”* (Urban FGD)

#### Disobedient women ensure other women receive insufficient care

In addition, participants identified that care providers often need to assist more than one woman at a time and that an uncooperative woman unfairly consumes more time as the providers try to convince her to co-operate, at the expense of having time to provide care for other women.

*“Some women want special attention and argue with the providers or refuse medically indicated procedure for no reason. Slapping or pinching is necessary for such selfish woman for consuming other women’s time.”* (Rural FGD)

A small number of participants discussed that yelling or shouting may be necessary to discipline women labelled as being “disobedient”. They expressed a viewpoint that providers have the right to yell or shout at women considered to be acting unreasonably.

*“Providers must raise their voice on disobedient women to ensure order in the delivery room.”* (Urban IDI)

In summary, participants did not consistently hold the same viewpoint across all scenarios, rather their perspectives changed depending on the unique characteristics of each scenario. While they generally considered disrespectful and abusive acts during childbirth as unacceptable, some acts presented in the scenarios were deemed necessary, under certain circumstances.

## Discussion

This study explored how women perceive acts often considered disrespectful and abusive by international standards [7]. Women were provided with scenarios and invited to discuss the circumstances under which the scenarios would be considered acceptable or unacceptable and when and why these circumstances might be acceptable. The results highlight the complexities of women’s responses in relation to disrespectful and abusive acts. This study offers new insights, as previous studies aimed at improving access to skilled birth care have focused more on availability of services and less on the perceptions of women as the recipients of care. More responsive and culturally acceptable care is possible when women’s perspectives and experiences are taken into consideration, potentially leading to an increase in service utilization, and better outcomes.

Women’s perspectives regarding disrespectful and abusive acts of maternity care providers in developing nations is best understood within the context of violence, limited health care resources and the societal hierarchies that have systematically disempowered women [17, 35–37]. For some of the women participants, disrespect and abuse is never acceptable in any of the scenarios. Women offering this perception tended to live in more urban areas and had higher levels of education.

Four of the examples of disrespect and abuse presented to participants were categorized as physical abuse; where a woman was pinched or slapped, forcefully held down to the delivery bed, her legs forcefully opened, and disallowance of preferred birthing position as reported by White Ribbon Alliance [7]. Women generally found these unacceptable although not all. A study from Guinea suggested that women provided justification for pinching or slapping in the belief that providers were using physical force in order to save the woman or baby’s life [38, 39]. Findings across both studies indicates that some women perceive there is a medical justification for slapping a woman, and that such behaviors are designed to “assist” the woman in pushing the baby out. Some participants in this study also considered the act of slapping as necessary to discipline “disobedient” women. Similarly, in a study from Nigeria, women believed that providers are justified to slap or pinch those “disobedient” women and discipline them [40].

The fact that a number of women in this study considered asking questions as time wasting, refusal of procedure as disobedience, and threatening harmless, indicates how societal hierarchies in Ethiopia have systematically disempowered women so that they feel they need to automatically obey the requests of the healthcare providers, while their own needs and preferences are ignored [41, 42]. Some of the women’s acceptance of disrespectful and abusive treatment by service providers during childbirth may be best understood within the context of violence in Ethiopia. For instance, the 2016 demographic and health survey reported that the majority of women (63%) and men (28%) have attitudes that justify wife beating [43]. Similar finding has been reported elsewhere in Africa [38].

Non-dignified care/ verbal abuse including women being shouted at or scolded, insulted, or blamed for negative pregnancy outcomes during facility-based childbirth is one of the most frequent types of disrespect and abuse women experience during childbirth [7, 44]. In this study, some women considered these behaviors unacceptable, however, a significant proportion of the participants provided justification for health professionals and considered yelling or shouting at women acceptable to save lives, help prevent complications or to discipline ‘disobedient’ women. Similar findings were reported in a study in Nigeria, Africa [40].

It is widely recognized that women should be given the information and support needed to freely make decisions for themselves and their newborns [45]. However, when women give birth in facilities in Ethiopia, informed consent is not always acquired or respected. Often, processes and procedures are not fully explained to women, as evidenced elsewhere [11, 14, 46, 47]. Internationally, and within Ethiopian health policy, performing procedures without prior knowledge of women is considered a violation of women’s rights [48]. Some women participants in this study believed providers can rightly perform procedures in emergency situations without seeking consent from the woman. Further, there were other participants who gave their right to decide their care to the health care providers. They expressed a viewpoint that consent is not a priority, and the provider is professional and knows what works best for them. Thus, they consider it is acceptable if the provider performs a procedure without telling them what is being done and why.

Acts of omission are also forms of disrespect and abuse and failure to recognize, protect, and fulfill a patient’s rights is a form of disrespect and abuse [49]. No participant considered refusal of care acceptable for any reason and under any circumstance. The majority of participants not only rejected withholding or refusing care but also condemned it. Some participants considered it as an act of evil similar to murder. Abandonment or denial of care is a prevalent form of disrespect and abuse in countries like Ethiopia and inadequate number of healthcare providers is the largest driving cause [50]. Ethiopia currently has inadequate skilled maternal health care providers to meet the essential needs of the population. According to the 2020 report of Federal Ministry of Health, the country had only 19,620 Midwives and does not meet the minimum threshold of health professionals to population ratio of 2.3 per 1000 population benchmark set by the WHO, for Sub-Saharan Africa [51].

Overall, this study used more comprehensive scenarios that reflect disrespect and abuse elements that are likely to occur in low-income countries. These build on and extend approaches used in previous studies conducted in Africa [11, 38, 40], with the aim of providing deeper explanation or enabling different, and at times conflicting, viewpoints to emerge. This study has found that normalisation of disrespect and abuse often experienced in private homes and workplaces has crossed over to health care. This has led to the current situation where women may accept and justify abusive behaviors of care providers, while ignoring their own needs and preferences.

This study was conducted in North Showa zone and findings in this study may not reflect the perceptions of women in other parts of Ethiopia. However, participants with diverse age, education and parity were involved in the study from rural and urban areas, which contributes to the diversity of perspectives included.

### Conclusion and recommendations

Women’s level of acceptance of disrespectful and abusive acts of care providers, varies from woman to woman, and from scenario to scenario, but is overall deeply rooted within the context of disrespect and abuse against women in Ethiopia and the societal hierarchies that have systematically disempowered women. Given the current pervasiveness of disrespect and abuse during childbirth care, policymakers, clinical managers and care providers must take these contextual and societal norms into account and devise a comprehensive clinical intervention involving health care providers and women that addresses these underlying perceptions. Women must be given safe platforms to identify, speak, and examine their care experiences, in discussions with clinicians and legislators to unravel the uncomfortable topic of disrespect and abuse that occurs during childbirth in health care facilities.

At a community programme level, there is a need to initiate respectful care awareness. The study’s findings regarding underlying gender-related conceptions and societal attitudes highlight the need for interventions and programs at a larger socio-political and communal level. Community involvement could include a range of activities such as information sharing, consultation, collaboration, and collective decision-making. The benefit of including the community in the ownership and sustainability of effective interventions is clearly established (230).

This study has identified areas for future research action. The women participants in this study gave birth at public health facilities. Future study could investigate the experiences of women who have used private health facilities or given birth at home to see if there are any disparities in their experiences.

In addition, many women in interviews expressed sadness, dissatisfaction or distress, indicating the emotional impact of disrespect and abuse. Future research could investigate further the negative impact of disrespect and abuse on women’s health, particularly from the perspective of those who have experienced abusive treatment in health institutions. Research could also investigate the negative effects disrespect and abuse of women has on babies, women’s partners and other family members.

The findings from the qualitative research methodology used in this study may not be generalisable to other settings (185). Therefore, large-scale studies with a representative sample size are needed to determine if the perspectives of women on disrespect and abuse expressed in this project can be generalised to the region and throughout Ethiopia as well as to similar countries. The potential for interventions to generate lasting improvements in labour and delivery experiences is perhaps the most intriguing area for further investigation. Moving beyond presenting well-documented problems, intervention research and evaluation of effectiveness is a crucial next step.

## Data Availability

The data that support the findings of this study are available from The University of Adelaide, but restrictions apply to the availability of these data, which were used under license for the current study, and so are not publicly available. Data are however available from Dr Janet Kelly upon reasonable request and with permission of The University of Adelaide.

## Declarations

### Authors information

Yohannes is a native Ethiopian and has worked in academia and health care sector for over a decade. Furthermore, his education (reproductive and child health) and research experience in maternity care have enabled him to understand the norms around childbirth in Ethiopia and traditions in the health care system. Janet is a nurse and midwife who has worked in urban and remote community health and research settings in Australia for over thirty years. Her research focuses on working collaboratively with community members, health and education professionals and students to improve access, equity, experiences and outcome of health care. Amy has been researching health care responses, violence against women and children and working with vulnerable groups in Australia for 15 years. Her research focuses on shifting the paradigm of care from disease and diagnosis-centeredness to person-centeredness and on prioritizing the lived experience of vulnerable groups in improving the delivery of care. Morgan is a nurse with expertise in qualitative research. She has lived and worked in rural areas of Australia and has a personal connection to Ethiopia.

## Abbreviations

D&A: Disrespect and Abuse
WHO: World Health Organization
